# Higher plasma high-mobility group box 1 levels are associated with incident cardiovascular disease and all-cause mortality in type 1 diabetes: a 12 year follow-up study

**DOI:** 10.1007/s00125-012-2622-1

**Published:** 2012-07-01

**Authors:** J. W. M. Nin, I. Ferreira, C. G. Schalkwijk, A. Jorsal, M. H. Prins, H.-H. Parving, L. Tarnow, P. Rossing, C. D. A. Stehouwer

**Affiliations:** 1 Department of Internal Medicine, Universiteitssingel 50, 6200 MD PO Box 616, Maastricht, the Netherlands; 2 Cardiovascular Research Institute Maastricht (CARIM), Maastricht University Medical Centre, Maastricht, the Netherlands; 3 Care and Public Health Research Institute (CAPHRI), Maastricht University Medical Centre, Maastricht, the Netherlands; 4 Department of Clinical Epidemiology and Medical Technology Assessment, Maastricht University Medical Centre, Maastricht, the Netherlands; 5 Steno Diabetes Center, Gentofte, Denmark; 6 Department of Endocrinology and Metabolism, Aarhus University Hospital, Aarhus, Denmark; 7 Department of Cardiology, Aarhus University Hospital, Aarhus, Denmark; 8 Department of Medical Endocrinology, Rigshospitalet, Copenhagen, Denmark; 9 Faculty of Health Science, Aarhus University, Aarhus, Denmark

**Keywords:** All-cause mortality, Cardiovascular disease, High-mobility group box 1, Type 1 diabetes mellitus

## Abstract

**Aims/hypothesis:**

This study aimed to investigate the associations of plasma levels of the pro-inflammatory cytokine high-mobility group box 1 (HMGB1) with incident cardiovascular disease (CVD) and all-cause mortality in patients with type 1 diabetes.

**Methods:**

We prospectively followed 165 individuals with diabetic nephropathy and 168 individuals with persistent normoalbuminuria who were free of CVD at study entry and in whom levels of HMGB1 and other cardiovascular risk factors were measured at baseline.

**Results:**

During the course of follow-up (median, 12.3 years [interquartile range, 7.8–12.5]), 80 patients died, 82 suffered a fatal (*n* = 46) and/or non-fatal (*n* = 53) CVD event. After adjustment for age, sex, case–control status and other risk factors, patients with higher levels of log_e_ HMGB1 had a higher incidence of fatal and non-fatal CVD and all-cause mortality: HR 1.55 (95% CI 0.94, 2.48) and HR 1.86 (95% CI 1.18, 2.93), respectively. Further adjustments for differences in markers of low-grade inflammation, endothelial and renal dysfunction and arterial stiffness did not attenuate these associations because plasma levels of HMGB1 were not independently associated with these variables.

**Conclusions/interpretation:**

In patients with type 1 diabetes, higher levels of plasma HMGB1 are independently associated with a higher risk of all-cause mortality and, to a lesser extent, with a higher incidence of CVD. Larger studies are needed to ascertain more definitely the role of HMGB1 in the development of vascular complications in diabetes.

**Electronic supplementary material:**

The online version of this article (doi:10.1007/s00125-012-2622-1) contains peer-reviewed but unedited supplementary material, which is available to authorised users.

## Introduction

The pathophysiological mechanisms linking hyperglycaemia to the development of cardiovascular complications in patients with diabetes mellitus are not completely clear. One proposed mechanism involves AGEs and the receptor for AGEs (RAGE) [1]. Besides AGEs, RAGE can also be activated by high-mobility group box 1 (HMGB1) [2].

Extracellular HMGB1 released from necrotic cells and/or from immune cells upon inflammatory stimulation functions as a pro-inflammatory cytokine, which elicits pro-inflammatory responses from macrophages and endothelial cells [2]. The extracellular effects of HMGB1 are mediated by its binding to RAGE, and also by its binding to the receptors of the toll-like receptor family [2]. HMGB1-induced production of inflammatory cytokines and adhesion molecules could contribute to low-grade inflammation (LGI), endothelial and renal dysfunction and arterial stiffening, all of which may partially explain the increased incidence of cardiovascular disease (CVD) in individuals with diabetes.

We therefore investigated, in a 12 year prospective study of patients with type 1 diabetes, first, the extent to which HMGB1 levels are associated with incident fatal and non-fatal CVD as well as all-cause mortality, and second the potential mediating role of markers of LGI, endothelial and renal dysfunction and/or arterial stiffness (i.e. pulse pressure).

## Methods

### Study design and population

We examined 199 patients with diabetic nephropathy (defined according to clinical criteria, i.e. persistent macroalbuminuria [>300 mg/24 h] in at least two out of three previous consecutive 24 h urine collections, the presence of diabetic retinopathy and the absence of other kidney or urinary tract disease) and 192 with persistent normoalbuminuria (i.e. urinary excretion rate <30 mg/24 h) who were recruited, in 1993, from the outpatient clinic at the Steno Diabetes Center for a prospective observational study, as described in detail elsewhere [1]. Briefly, all patients were followed up to the last visit to the Center, until 1 September 2006, death (*n* = 82) or emigration (*n* = 3). The study was approved by the local ethics committee in accordance with the Helsinki Declaration, and all patients gave their informed written consent.

### Main determinant

Baseline levels of plasma HMGB1 were measured in duplicate with a commercially available enzyme-linked immunosorbent assay kit (Shino-Test Corporation, Tokyo, Japan) and the intra- and inter-assay coefficients of variation were 4.0% and 11.0%, respectively.

### Outcomes

Study outcomes were a combined endpoint of fatal and non-fatal CVD, and all-cause mortality [1].

### Statistical analyses

All analyses were performed using SPSS version 15.0 (SPSS, Chicago, IL, USA). Variables with a skewed distribution were log_e_ transformed prior to further analyses. Comparisons of baseline characteristics between groups were performed with Student’s *t* or *χ* ^2^ tests, as appropriate.

We used linear regression to investigate the cross-sectional associations between HMGB1 and other cardiovascular risk factors, and Cox proportional hazards regression to investigate the associations of plasma HMGB1 with incident CVD and all-cause mortality. These analyses were adjusted for age, sex, duration of diabetes and case–control status, and adjusted further for other cardiovascular risk factors and use of medication (i.e. potential confounders). Other adjustments included markers of LGI, endothelial dysfunction, renal dysfunction and pulse pressure (PP) (i.e. potential mediators) to ascertain the extent to which any such marker(s) could attenuate (i.e. explain) the strength of the association between HMGB1 and study endpoints.

A detailed description of measurement of study outcomes, potential confounders and mediators is given in the electronic supplementary material (ESM) Methods.

## Results

We excluded 17 patients for whom follow-up data were not obtained, ten with missing data on baseline biomarker levels, seven with end-stage renal failure and 24 with prior CVD at baseline. Results reported herein thus refer to 333 patients (165 with nephropathy and 168 with persistent normoalbuminuria at baseline).

**Fig. 1:**
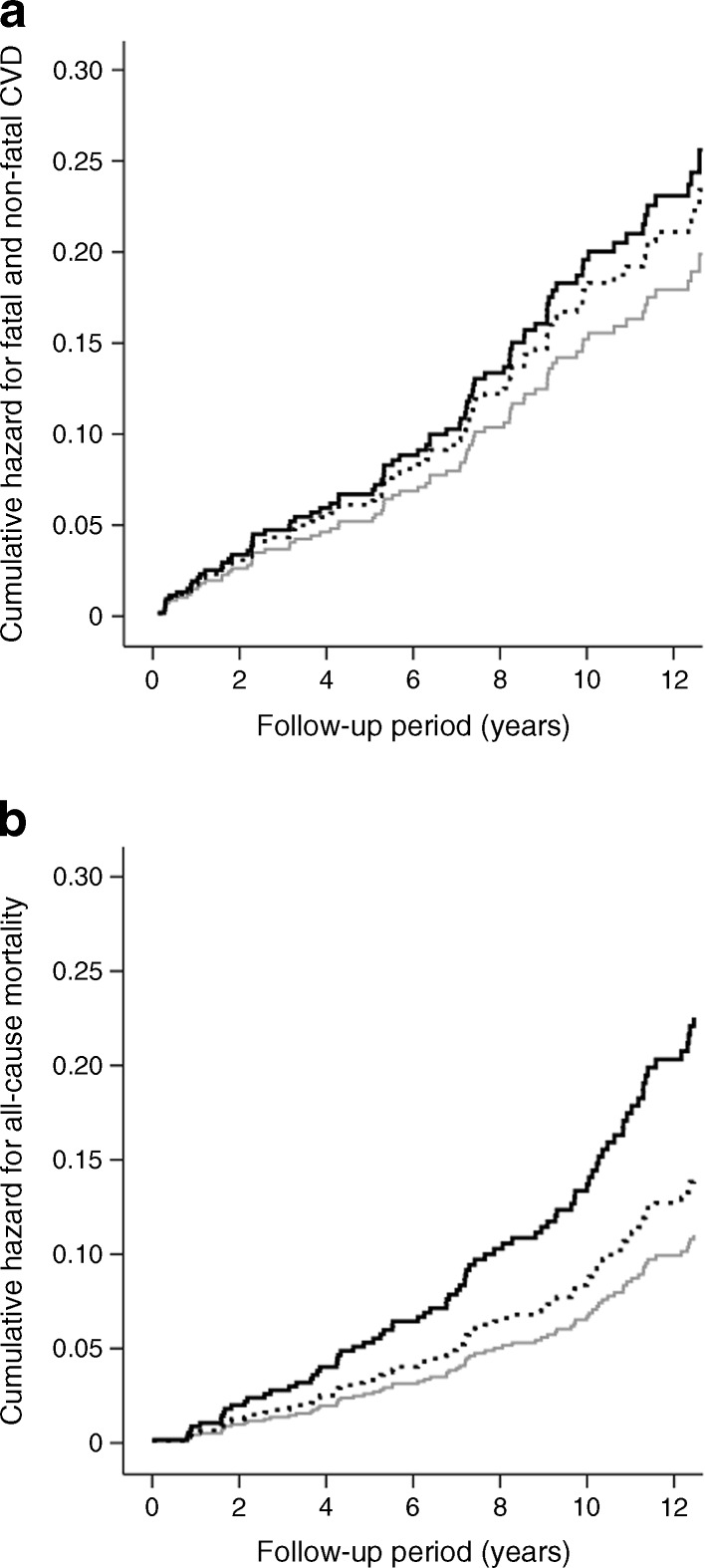
Cumulative hazard for fatal and non-fatal CVD (**a**) and all-cause mortality (**b**) across tertiles of plasma HMGB1. Data are adjusted for age, sex, case–control status, duration of diabetes, HbA_1c_, smoking status, MAP, BMI, total cholesterol, use of anti-hypertensive treatment and continuation of medication use at baseline examination. Compared with patients in the lowest tertile of HMGB1 (grey line), those in the middle (dotted line) and highest (black line) tertiles had increased risk for fatal and non-fatal CVD (HR 1.18 [95% CI 0.68, 2.05] and HR 1.29 [0.73, 2.28]), respectively, *p* for trend = 0.337 and all-cause mortality (HR 1.28 [95% CI 0.70, 2.33] and 2.05 [1.14, 3.67], respectively, *p* for trend = 0.018)

During the course of follow-up (median: 12.3 years [interquartile range (IQR) 7.8–12.5]), 80 individuals (24.0%) died, 82 (24.6%) suffered a fatal (*n* = 46) and/or non-fatal (*n* = 53) CVD event. Individuals with incident CVD or who died in the course of follow-up had a more adverse atherosclerotic risk profile at baseline (ESM Table 1), although their median levels of HMGB1 (unadjusted for study covariates) did not differ significantly from the levels of those who remained free of CVD or were still alive: 0.77 ng/ml (IQR 0.62–0.95) vs 0.77 (IQR 0.61–1.03) and 0.78 (IQR 0.64–0.99) vs 0.77 (0.60–1.01), respectively. However, after adjustments for age, sex, duration of diabetes, case–control status, HbA_1c_, BMI, smoking status, total cholesterol, mean arterial pressure (MAP) and use of medication, their baseline levels of HMGB1 were indeed increased such that higher levels of HMGB1 (log_e_-transformed) were associated with a higher incidence of fatal and non-fatal CVD (HR 1.55 [95% CI 0.96, 2.51]) and all-cause mortality (HR 1.86 [95% CI 1.18, 2.93]) (Table 1, model 5, Fig. 1a, b). The magnitude of the associations between HMGB1 and study outcomes increased and were significant only after covariate adjustments, due to net negative confounding mainly by age, HbA_1c_ and total cholesterol, all of which were associated inversely with HMGB1 but positively with study outcomes (ESM Tables 2 and 3).

**Table 1:** Associations between plasma log_e_ HMGB1 and incident fatal and non-fatal CVD and all-cause mortality (*n* = 333)

Model: adjustments	Fatal and non-fatal CVD			All-cause mortality		
	HR	95% CI	*p* value	HR	95% CI	*p* value
1: –	1.11	0.68, 1.79	0.689	1.34	0.84, 2.15	0.221
2: age and sex	1.27	0.78, 2.09	0.323	1.62	1.00, 2.62	0.049
3: model 2 + case–control status, duration of diabetes and HbA_1c_	1.37	0.86, 2.20	0.186	1.65	1.06, 2.57	0.028
4: model 3 + MAP, BMI, smoking status and total cholesterol	1.47	0.91, 2.36	0.113	1.72	1.08, 2.74	0.022
5: model 4 + use of anti-hypertensive medication^a^	1.55	0.96, 2.51	0.073	1.86	1.18, 2.93	0.008
6a: model 5 + LGI score^b^	1.50	0.93, 2.43	0.095	1.81	1.15, 2.87	0.011
6b: model 5 + endothelial dysfunction score^c^	1.55	0.96, 2.51	0.073	1.81	1.14, 2.87	0.012
6c: model 5 + eGFR_MDRD_ and log_e_ UAE	1.54	0.96, 2.45	0.071	1.80	1.14, 2.84	0.011
6d: model 5 + PP	1.49	0.92, 2.42	0.105	1.80	1.14, 2.85	0.012
7a: model 5 + AGE score^d^	1.52	0.94, 2.46	0.087	1.78	1.12, 2.82	0.014
7b: model 5 + log_e_ sRAGE^e^	1.59	0.97, 2.61	0.063	1.86	1.17, 2.97	0.009

HR per each unit increase in log_e_ HMGB1 levels at baseline

^a^Includes patients discontinuing medication before the baseline assessment

^b^Score comprising levels of C-reactive protein, secreted phospholipase A2 and IL-6

^c^Score comprising levels of soluble vascular cell adhesion molecule-1 (sVCAM-1) and soluble intercellular adhesion molecule-1

^d^Score comprising levels of protein-bound *N* ^ε^-(carboxyethyl)lysine, *N* ^ε^-(carboxymethyl)lysine and pentosidine

^e^Analyses confined to 331 individuals because of missing data for two individuals

eGFR, estimated GFR; MDRD, Modification of Diet in Renal Disease equation; UAE, urinary albumin excretion rate

The adverse associations between HMGB1 and study outcomes were not appreciably attenuated after further adjustments for markers of LGI, endothelial and renal dysfunction and PP (models 6a–d), because HMGB1 was not independently associated with these variables (ESM Table 4). Further adjustment for AGEs or soluble RAGE (sRAGE) did not appreciably change the effect estimates either (models 7a,b).

### Additional analyses

We also investigated the associations between HMGB1 and study outcomes stratified by case–control status. The effect estimates seemed stronger in the group of patients with normoalbuminuria (HR 4.17 [95% CI 0.75, 2.17] for fatal and non-fatal CVD and HR 7.64 [95% CI 1.91, 30.60] for all-cause mortality) than nephropathy (1.28 [95% CI 0.75, 2.17] and 1.59 [95% CI 0.98, 2.61], respectively), but did not differ significantly between the groups (*p* interactions = 0.177 and 0.142, respectively). These data should be interpreted with caution and may not justify an interpretation of true differences between the groups because these analyses were underpowered (only 20 CVD events and 17 all-cause deaths in the normoalbuminuria group).

## Discussion

The main findings of this study are that, in patients with type 1 diabetes, and after adjustments for confounders, higher levels of plasma HMGB1 are associated with a higher incidence of all-cause mortality and also, though to a lesser extent, fatal and non-fatal CVD.

These findings are in agreement with three studies that have reported positive associations of HMGB1 with coronary artery disease [3, 4], heart failure [5] and mortality related to heart disease [5] in patients with and without type 2 diabetes, though these were limited by their cross-sectional study design [3–5] or short follow-up period [5]. The adverse role of elevated HMGB1 levels is supported by observations at the molecular level showing that fatty streaks and fibrofatty lesions contain more macrophages with cytoplasmic and nucleic HMGB1 compared with normal intima [6], and that HMGB1 is also expressed by activated vascular smooth muscle cells in more advanced atherosclerotic lesions [7]. Furthermore, neutralising HMGB1 attenuated the development of atherosclerosis in an animal model of atherosclerosis [8]. HMGB1 has been linked not only to diabetes [4] and CVD [3–5], but also to inflammatory diseases and cancer [9], which may explain the stronger association with all-cause mortality than with CVD observed in the present study.

While investigating the associations between HMGB1 and traditional risk factors we found positive associations with smoking but inverse associations with age, HbA_1c_ and cholesterol. Indeed, a net negative confounding effect explained why, after adjustments for these (and other) confounders, the adverse associations between HMGB1 and study outcomes were strengthened and became statistically significant. The reasons for the inverse associations between HMGB1 and some risk factors are unclear and need to be further investigated. Still, our study illustrates the importance of accounting for confounding when examining the potential value of a biomarker in outcome prediction. We did not find independent associations between HMGB1 and LGI, endothelial and renal dysfunction or PP, mechanisms that could explain the increased CVD and mortality risk associated with HMGB1. Given that we examined a selection of biomarkers of these processes, we cannot fully rule out their potential mediating role, but our findings suggest that these pathophysiological mechanisms and HMGB1 may constitute distinct pathways leading to poorer outcome in these patients.

There are limitations to our study. First, measures of HMGB1 and other biomarkers were taken at baseline only. Second, an inter-assay variation lower than 11%, as obtained in our HMGB1 measures, may enable more precise estimates of the associations examined. Third, we have recently shown that in patients with type 1 diabetes (EURODIAB study) serum HMGB1 was not associated with prevalent CVD [10]. Apart from the difference in study design (cross-sectional vs prospective), the apparent discrepancy with the positive association between plasma HMGB1 and incident CVD observed in the present study raises the possibility that measures obtained in serum vs plasma may not represent the same pool of HMGB1. In addition, it is not known how plasma or serum levels of HMGB1 relate to intracellular levels.

In conclusion, higher levels of plasma HMGB1 may play a role in the development of CVD and high mortality risk in type 1 diabetes and may constitute a specific target for treatment in these patients. However, further studies are needed to clarify the determinants of HMGB1 and the inter-relationship between plasma levels of HMGB1 and its various receptors and their associations with CVD.

## Electronic supplementary material

Below is the link to the electronic supplementary material.

ESM Methods (PDF 55 kb)

ESM Table 1 (PDF 125 kb)

ESM Table 2 (PDF 63 kb)

ESM Table 3 (PDF 42 kb)

ESM Table 4 (PDF 158 kb)

## Abbreviations

CVD: Cardiovascular disease
HMGB1: High-mobility group box 1
LGI: Low-grade inflammation
MAP: Mean arterial pressure
PP: Pulse pressure
RAGE: Receptor for AGEs
sRAGE: Soluble receptor for AGEs
