# Supplementary material for: Higher plasma high-mobility group box 1 levels are associated with incident cardiovascular disease and all-cause mortality in type 1 diabetes: a 12 year follow-up study
Source: Diabetologia. 2012 Jul 1;55(9):2489–93. doi: 10.1007/s00125-012-2622-1 (PMC3411294; doi:10.1007/s00125-012-2622-1)
Supplement: Supplementary file 1 — (PDF 55 kb) [file 125_2012_2622_MOESM1_ESM.pdf]

Electronic supplementary material - Methods

Higher plasma high-mobility group box 1 levels are associated with incident cardiovascular disease and all-cause mortality in type 1 diabetes: a 12 year follow-up study

J. W. M. Nin[1,2,3], I. Ferreira[1,2,3,4], C. G. Schalkwijk[1,2], A. Jorsal[5,6,7], M. H. Prins[3,4], H.-H. Parving[8,9], L. Tarnow[5], P. Rossing[5] and C. D. A. Stehouwer[1,2]

1. Department of Internal Medicine, Universiteitssingel 50, 6200 MD, PO Box 616, Maastricht, the Netherlands
2. Cardiovascular Research Institute Maastricht (CARIM), Maastricht University Medical Centre, the Netherlands
3. Care and Public Health Research Institute (CAPHRI), Maastricht University Medical Centre, the Netherlands
4. Department of Clinical Epidemiology and Medical Technology Assessment, Maastricht University Medical Centre, the Netherlands
5. Steno Diabetes Center, Gentofte, Denmark
6. Department of Endocrinology and Metabolism, Aarhus University Hospital, Aarhus, Denmark
7. Department of Cardiology, Aarhus University Hospital, Aarhus, Denmark
8. Department of Medical Endocrinology, Rigshospitalet, Copenhagen, Denmark
9. Faculty of Health Science, Aarhus University, Aarhus, Denmark

Corresponding author: J. W. M. Nin, Department of Internal Medicine, Universiteitssingel 50, 6200 MD, PO Box 616, Maastricht, the Netherlands
email j.nin@maastrichtuniversity.nl

Methods

Baseline investigations

All investigations were performed in the morning after an overnight fast. No antihypertensive medication was ever prescribed in 24% of patients with nephropathy and 88% of the normoalbuminuric patients. All of the remaining patients were asked to stop their antihypertensive and diuretic treatment 8 days before the examination and 84.1% of the patients complied with this request.

Haemoglobin A1c (HbA1c) was measured by high performance liquid chromatography (DIAMAT Analyzer, Bio-Rad, Hercules, Calif., USA). Arterial blood pressure was measured twice with an appropriate cuff size following at least 10 minutes rest in the supine position. Mean arterial pressure (MAP) was calculated as [systolic blood pressure + (2*diastolic blood pressure)]/3. Pulse pressure (PP) was calculated by subtracting the diastolic from the systolic blood pressure, and used as a marker of arterial stiffness [1]. BMI was calculated by dividing weight by height squared. Serum total cholesterol was determined enzymatically using CHOD-PAP reagents from Boehringer-Mannheim GmbH (Mannheim, Germany). Urinary albumin excretion (UAE) was measured by an enzyme immunoassay from 24-h urine collections. Serum creatinine concentration was assessed by a kinetic Jaffé method. In all patients glomerular filtration rate (eGFR) was estimated according to the short Modification of Diet in Renal Disease equation (MDRD) [2]. Patients were interviewed using the WHO cardiovascular questionnaire. Individuals were categorised into three groups according to their smoking status as never, former or current smokers.

Analyses of the biomarkers were done on frozen (-80°C) samples and were performed at a central laboratory by C.G.S. High-sensitivity C-reactive protein (hsCRP) and secreted phospholipase A2 (sPLA2) were determined by ELISA as described previously [3]. Commercially available ELISA kits were used for measurements of plasma soluble vascular cell adhesion molecule-1 (sVCAM-1), soluble intercellular adhesion molecule-1 (sICAM-1) and interleukin-6 (IL-6) (Quantikine High Sensitivity; R&D Systems, Oxon, U.K.). The intra- and inter-assay CVs of these immunoassays were all <8%. Protein-bound N^{ε}-(carboxyethyl)lysine (CEL) and N^{ε}-(carboxymethyl)lysine (CML) were determined in plasma by stable-isotope dilution tandem mass spectrometry [4]. Protein-bound pentosidine levels were determined in plasma by a single step reversed phase based high-performance liquid chromatography separation with fluorescent detection. The intra- and inter-assay CVs were 7.3% and 2.0% for CEL, 3.5% and 3.6% for CML, and 2.7% and 2.8% for pentosidine, respectively. Plasma levels of soluble receptor for advanced glycation endproducts (sRAGE) were measured in duplicate using a commercially available ELISA kit (Quantikine; R&D systems, Minneapolis, MN, USA) and the intra- and inter-assay CV values were 2% and 17.5%, respectively.

An overall low-grade inflammation score was calculated by averaging the z scores of Ln-IL-6, Ln-CRP, sICAM-1 and Ln-sPLA2; likewise, an overall endothelial dysfunction score was calculated by averaging the z scores of sVCAM-1 and sICAM-1 and an overall advanced glycation endproducts (AGEs) score was calculated by averaging the z-scores of CML, CEL and pentosidine.

Study endpoints

All patients were traced through the national register during autumn 2006. If a patient had died before 1 September 2006, the date of death was recorded and information on the primary cause of death was obtained from the death certificate, which was reviewed by two

independent observers. Additional available information from necropsy reports was also included. All deaths were classified as cardiovascular unless an unequivocal non-cardiovascular cause was established. In all patients alive at the end of follow-up, non-fatal cardiovascular events were retrieved from their patient files from Steno Diabetes Center and/or other hospital records. The primary study outcome was a combined endpoint of fatal and non-fatal cardiovascular disease (i.e. myocardial infarction, percutaneous coronary intervention, coronary bypass grafting, amputation due to ischaemia, vascular surgery for peripheral atherosclerotic disease and stroke), and the secondary outcome was all-cause mortality [4].

References

[1] Stehouwer CD, Henry RM, Ferreira I (2008) Arterial stiffness in diabetes and the metabolic syndrome: a pathway to cardiovascular disease. Diabetologia 51: 527-539

[2] Levey AS, Bosch JP, Lewis JB, Greene T, Rogers N, Roth D (1999) A more accurate method to estimate glomerular filtration rate from serum creatinine: a new prediction equation. Modification of Diet in Renal Disease Study Group. Ann Intern Med 130: 461-470

[3] De Jager J, Kooy A, Lehert P, et al. (2005) Effects of short-term treatment with metformin on markers of endothelial function and inflammatory activity in type 2 diabetes mellitus: a randomised, placebo-controlled trial. J Intern Med 257: 100-109

[4] Nin JW, Jorsal A, Ferreira I, et al. (2011) Higher plasma levels of advanced glycation end products are associated with incident cardiovascular disease and all-cause mortality in type 1 diabetes: a 12-year follow-up study. Diabetes Care 34: 442-447
