# Supplementary material for: Higher plasma high-mobility group box 1 levels are associated with incident cardiovascular disease and all-cause mortality in type 1 diabetes: a 12 year follow-up study
Source: Diabetologia. 2012 Jul 1;55(9):2489–93. doi: 10.1007/s00125-012-2622-1 (PMC3411294; doi:10.1007/s00125-012-2622-1)
Supplement: Supplementary file 2 — (PDF 125 kb) [file 125_2012_2622_MOESM2_ESM.pdf]

ESM Table 1 Baseline characteristics according to the occurrence of cardiovascular events and of death during follow-up

	Fatal or non-fatal CVD event N=82	No CVD event N=251	*p* value	Died at follow-up N=80	Alive at follow-up N=253	*p* value
Age (yrs)	44.6 (9.2)	40.4 (9.6)	0.001	45.5 (10.1)	40.2 (9.2)	<0.001
Sex: male (%)	61.0	60.6	0.946	68.8	58.1	0.115
Nephropathy (%)	75.6	41.0	<0.001	76.8	40.6	<0.001
Duration of diabetes (yrs)	30.8 (8.7)	26.8 (7.5)	<0.001	30.2 (10.0)	27.0 (7.2)	0.008
HbA1c (%)	9.6 (1.5)	8.8 (1.4)	<0.001	9.6 (1.5)	8.8 (1.3)	<0.001
Diastolic blood pressure (mmHg)	85 (13)	79 (12)	<0.001	87 (13)	79 (11)	<0.001
Systolic blood pressure (mmHg)	157 (24)	135 (20)	<0.001	160 (24)	135 (19)	<0.001
Mean arterial pressure (mmHg)	109 (14)	98 (13)	<0.001	111 (14)	97 (12)	<0.001
Pulse pressure (mmHg)	72 (21)	57 (15)	<0.001	73 (21)	57 (15)	<0.001
BMI (kg/m^2)	23.9 (3.3)	23.9 (2.8)	0.957	23.8 (3.2)	23.9 (2.9)	0.655
Smoking: never/former/current (%)	29.3/18.3/52.4	37.5/17.5/45.0	0.378	26.3/16.3/57.4	38.3/18.2/43.5	0.073
Total cholesterol (mmol/l)	5.71 (1.12)	5.00 (1.13)	<0.001	5.81 (1.13)	4.97 (1.10)	<0.001
RAAS inhibitors: yes (%)	51.2	19.5	<0.001	51.3	19.8	<0.001
Other antihypertensive treatment: yes (%)	64.6	28.2	<0.001	70.0	26.9	<0.001
Discontinuation of medication at baseline(%)	67.1	89.6	<0.001	68.8	88.9	<0.001
Retinopathy: no/simplex/proliferative (%)	8.5/36.6/54.9	21.1/45.0/33.9	0.001	6.3/37.5/56.2	21.7/44.7/33.6	<0.001
C-reactive protein (mg/l)	1.63 [0.73-3.24]	0.97 [0.42-2.27]	0.008	1.45 [0.64-3.36]	1.02 [0.44-2.19]	0.016
Interleukin-6 (pg/ml)	2.17 [1.53-3.43]	1.49 [0.98-2.34]	<0.001	2.38 [1.79-3.91]	1.44 [1.00-2.21]	<0.001
Secreted phospholipase A2 (µg/ml)	4.40 [2.80-6.95]	4.00 [2.70-6.30]	0.313	4.05 [2.80-6.45]	4.00 [2.70-6.60]	0.844
Low-grade inflammation score	0.24 (0.63)	-0.08 (0.76)	0.001	0.23 (0.69)	-0.07 (0.75)	0.001
sICAM-1 (ng/ml)	780 (254)	726 (273)	0.115	817 (281)	715 (261)	0.003
sVCAM-1 (ng/ml)	1051 (273)	982 (348)	0.102	1105 (350)	966 (320)	0.001
Endothelial dysfunction score	0.15 (0.70)	-0.05 (0.81)	0.041	0.30 (0.84)	-0.10 (0.75)	<0.001
N^{ε}-(carboxyethyl)lysine (µmol/l)	1.02 (0.29)	0.92 (0.19)	0.007	1.03 (0.30)	0.92 (0.18)	0.004
N^{ε}-(carboxymethyl)lysine (µmol/l)	3.59 (1.16)	3.53 (0.84)	0.657	3.55 (1.35)	3.55 (0.75)	0.990
Pentosidine (pmol/mg)	50.7 [34.5-71.8]	40.8 [34.2-48.9]	0.005	51.8 [33.4-71.9]	41.0 [34.4-48.9]	0.008
Advanced glycation endproducts score	0.24 (1.22)	-0.08 (0.78)	0.030	0.24 (1.36)	-0.08 (0.71)	0.048
sRAGE (ng/ml)*	1.04 [0.80-1.41]	0.97 [0.72-1.36]	0.041	1.13 [0.83-1.52]	0.96 [0.72-1.31]	0.001
Estimated GFR_{MDRD} (ml/min/1.73m^2)	65 (29)	87 (21)	<0.001	65 (27)	87 (22)	<0.001
Urinary albumin excretion rate (mg/24h)	658 [34-1983]	16 [7-495]	<0.001	762 [100-2030]	15 [7-451]	<0.001

Data are means (SD), medians [inter-quartile range] or percentages, as appropriate. CVD, cardiovascular disease; RAAS-inhibitors, renin-angiotensin-aldosterone system inhibitors; sICAM-1, soluble intercellular adhesion molecule-1; sVCAM-1, soluble vascular cell adhesion molecule-1; sRAGE, soluble receptor for advanced glycation endproducts; estimated GFR_{MDRD}, estimated glomerular filtration rate by abbreviated modification of diet in renal disease equation.
* analyses confined to 331 subjects because of missing data in 2 individuals
