# Supplementary material for: Higher plasma high-mobility group box 1 levels are associated with incident cardiovascular disease and all-cause mortality in type 1 diabetes: a 12 year follow-up study
Source: Diabetologia. 2012 Jul 1;55(9):2489–93. doi: 10.1007/s00125-012-2622-1 (PMC3411294; doi:10.1007/s00125-012-2622-1)
Supplement: Supplementary file 3 — (PDF 63 kb) [file 125_2012_2622_MOESM3_ESM.pdf]

ESM Table 2 Associations between cardiovascular risk factors and $\log_e$ HMGB1 (N=333)

	Model 1: univariate			Model 2: age and sex-adjusted			Model 3: Fully adjusted		
Independent variable	β	95% CI	*p* value	β	95% CI	*p* value	β	95% CI	*p* value
Age (per SD)	-0.15	-0.26; -0.04	0.006	-0.15	-0.26; -0.05	0.005	-0.11	-0.24; 0.03	0.131
Female sex	-0.20	-0.42; 0.02	0.079	-0.21	-0.43; 0.01	0.061	-0.16	-0.38; 0.07	0.166
Nephropathy at baseline	0.02	-0.20; 0.23	0.875	-0.02	-0.24; 0.19	0.843	0.16	-0.14; 0.46	0.303
Duration of diabetes (per SD)	-0.12	-0.23; -0.02	0.024	-0.04	-0.17; 0.10	0.578	-0.01	-0.14; 0.12	0.867
HbA1c (per SD)	-0.12	-0.23; -0.02	0.024	-0.12	-0.22; -0.01	0.034	-0.12	-0.23; 0.00	0.047
Mean arterial pressure (per SD)	0.02	-0.09; 0.13	0.727	0.02	-0.09; 0.13	0.699	0.11	-0.02; 0.24	0.110
BMI (per SD)	-0.10	-0.21; 0.01	0.069	-0.10	-0.20; 0.01	0.079	-0.07	-0.18; 0.04	0.210
Smoking status									
former vs. never	-0.13	-0.44; 0.18	0.397	-0.02	-0.16; 0.12	0.761	-0.02	-0.34; 0.29	0.881
current vs. never	0.29	0.05; 0.53	0.017	0.30	0.07; 0.54	0.012	0.33	0.09; 0.57	0.007
Total cholesterol (per SD)	-0.16	-0.26; -0.05	0.004	-0.14	-0.25; -0.04	0.009	-0.16	-0.28; -0.04	0.008
RAAS inhibitors	-0.06	-0.31; 0.18	0.603	-0.09	-0.33; 0.15	0.450	-0.04	-0.35; 0.27	0.796
Other anti-hypertensive treatment	-0.16	-0.38; 0.07	0.163	-0.11	-0.34; 0.11	0.315	-0.10	-0.41; 0.20	0.508
Discontinuation of medication at baseline	0.16	-0.14; 0.45	0.295	0.11	-0.18; 0.40	0.454	0.04	-0.29; 0.37	0.822

β, standardised regression coefficient: indicates change in $\log_e$ HMGB1 (in SD) per SD increase in independent variable.
