# Supplementary material for: Higher plasma high-mobility group box 1 levels are associated with incident cardiovascular disease and all-cause mortality in type 1 diabetes: a 12 year follow-up study
Source: Diabetologia. 2012 Jul 1;55(9):2489–93. doi: 10.1007/s00125-012-2622-1 (PMC3411294; doi:10.1007/s00125-012-2622-1)
Supplement: Supplementary file 4 — (PDF 42 kb) [file 125_2012_2622_MOESM4_ESM.pdf]

ESM Table 3 Associations between study covariates with incident fatal and non-fatal CVD and all cause mortality

	Model 1: univariate			Model 2: Age and sex-adjusted			Model 3: Fully adjusted		
Independent variable	HR	95% CI	*p* value	HR	95% CI	*p* value	HR	95% CI	*p* value
Outcome: fatal and non-fatal CVD									
Age (per SD)	1.43	1.16; 1.78	0.001	1.43	1.15; 1.77	0.001	1.31	0.99; 1.75	0.061
Female sex	0.88	0.56; 1.37	0.563	0.92	0.59; 1.44	0.730	0.92	0.56; 1.50	0.729
Nephropathy at baseline	3.62	2.18; 6.00	<0.001	4.06	2.44; 6.76	<0.001	1.36	0.67; 2.74	0.392
Duration of diabetes (per SD)	1.47	1.21; 1.79	<0.001	1.34	1.05; 1.70	0.017	1.14	0.89; 1.47	0.300
HbA1c (per SD)	1.47	1.21; 1.79	<0.001	1.52	1.23; 1.86	<0.001	1.19	0.95; 1.50	0.126
Mean arterial pressure (per SD)	1.93	1.58; 2.35	<0.001	1.91	1.55; 2.34	<0.001	1.51	1.17; 1.95	0.001
BMI (per SD)	0.99	0.79; 1.23	0.922	0.96	0.77; 1.20	0.721	0.89	0.71; 1.11	0.288
Smoking status									
former vs. never	1.10	0.57; 2.12	0.768	0.89	0.46; 1.73	0.735	1.05	0.53; 2.10	0.893
current vs. never	1.44	0.87; 2.37	0.155	1.40	0.85; 2.30	0.192	1.62	0.95; 2.75	0.074
Total cholesterol (per SD)	1.68	1.39; 2.04	<0.001	1.62	1.34; 1.98	<0.001	1.25	0.99; 1.58	0.062
RAAS inhibitors	3.26	2.11; 5.03	<0.001	3.34	2.16; 5.17	<0.001	1.14	0.65; 1.98	0.654
Other anti-hypertensive treatment	3.69	2.34; 5.82	<0.001	3.64	2.30; 5.76	<0.001	1.48	0.80; 2.71	0.210
Discontinuation of medication	0.28	0.18; 0.45	<0.001	0.28	0.17; 0.44	<0.001	0.50	0.29; 0.86	0.013
Outcome: all-cause mortality									
Age (per SD)	1.60	1.29; 198	<0.001	1.57	1.27; 1.95	<0.001	1.72	1.30; 2.26	<0.001
Female sex	0.66	0.41; 1.06	0.088	0.70	0.44; 1.13	0.143	0.69	0.41; 1.14	0.148
Nephropathy at baseline	4.45	2.60; 7.61	<0.001	1.64	3.02; 8.87	<0.001	1.42	0.69; 2.94	0.340
Duration of diabetes (per SD)	1.43	1.16; 1.75	0.001	1.20	0.94; 1.53	0.154	0.95	0.73; 1.24	0.711
HbA1c (per SD)	1.57	1.29; 1.91	<0.001	1.67	1.35; 2.06	<0.001	1.31	1.04; 1.66	0.021
Mean arterial pressure (per SD)	2.23	1.82; 2.73	<0.001	2.16	1.76; 2.67	<0.001	1.85	1.42; 2.41	<0.001
BMI (per SD)	0.93	0.74; 1.17	0.528	0.87	0.68; 1.10	0.238	0.74	0.59; 0.92	0.007
Smoking status									
former vs. never	1.27	0.63; 2.53	0.504	0.89	0.43; 1.80	0.736	0.99	0.48; 2.06	0.977
current vs. never	1.77	1.06; 2.96	0.031	1.65	0.98; 2.76	0.060	1.62	0.94; 2.79	0.081
Total cholesterol (per SD)	1.74	1.43; 2.10	<0.001	1.65	1.36; 2.00	<0.001	1.24	0.98; 1.57	0.076
RAAS inhibitors	3.23	2.09; 5.02	<0.001	3.32	2.13; 5.17	<0.001	0.82	0.47; 1.43	0.484
Other anti-hypertensive treatment	4.94	3.06; 7.98	<0.001	5.09	3.14; 8.25	<0.001	2.31	1.24; 4.32	0.009
Discontinuation of medication	0.33	0.20; 0.53	<0.001	0.32	0.20; 0.51	<0.001	0.65	0.38; 1.14	0.134

HR, hazard-ratio per SD increase in independent variable
