# Supplementary material for: Higher plasma high-mobility group box 1 levels are associated with incident cardiovascular disease and all-cause mortality in type 1 diabetes: a 12 year follow-up study
Source: Diabetologia. 2012 Jul 1;55(9):2489–93. doi: 10.1007/s00125-012-2622-1 (PMC3411294; doi:10.1007/s00125-012-2622-1)
Supplement: Supplementary file 5 — (PDF 158 kb) [file 125_2012_2622_MOESM5_ESM.pdf]

ESM Table 4 Associations between plasma $\log_e$ HMGB1 and markers of low-grade inflammation, endothelial and renal dysfunction, advanced glycation endproducts, and pulse pressure (n=333)

	Model 1			Model 2		
Dependent variable	β	95% CI	*p* value	β	95% CI	*p* value
$\log_e$ C-reactive protein	0.05	-0.06; 0.16	0.354	0.06	-0.05; 0.17	0.266
$\log_e$ interleukin-6	0.11	0.00; 0.21	0.049	0.08	-0.03; 0.19	0.141
$\log_e$ secreted phospholipase A2	0.04	-0.06; 0.14	0.422	0.02	-0.08; 0.13	0.668
Low-grade inflammation score	0.07	-0.01; 0.14	0.097	0.05	-0.02; 0.13	0.173
Soluble vascular cell adhesion molecule-1	-0.02	-0.13; 0.08	0.677	-0.02	-0.12; 0.09	0.735
Soluble intercellular adhesion molecule-1	0.15	0.04; 0.26	0.007	0.10	-0.01; 0.20	0.071
Endothelial dysfunction score	0.06	-0.02; 0.15	0.134	0.04	-0.05; 0.12	0.371
Baseline $eGFR_{MDRD}$	0.03	-0.06; 0.12	0.487	0.00	-0.08; 0.09	0.938
$\log_e$ urinary albumin excretion rate	0.01	-0.03; 0.05	0.668	0.02	-0.03; 0.06	0.434
Pulse pressure	0.03	-0.06; 0.13	0.485	0.03	-0.05; 0.12	0.442
N^{ε}-(carboxyethyl)lysine	0.08	-0.03; 0.18	0.158	0.08	0.03; 0.18	0.148
N^{ε}-(carboxymethyl)lysine	0.08	-0.03; 0.18	0.138	0.08	-0.03; 0.18	0.135
$\log_e$ pentosidine	0.00	-0.11; 0.10	0.946	0.01	-0.09; 0.11	0.878
Advanced glycation endproducts score	0.05	-0.05; 0.15	0.300	0.05	-0.04; 0.15	0.252
$\log_e$ sRAGE	-0.05	-0.16; 0.06	0.361	-0.06	-0.17; 0.05	0.285

β, standardised regression coefficient: indicates change in dependent variable (in SD) per 1 SD increase in $\log_e$ HMGB1.
Model 1, adjusted for age, sex, case-control status, duration of diabetes and HbA1c;
Model 2, model 1 + additional adjustments for mean arterial pressure, BMI, smoking status, total cholesterol, use of renin-angiotensin-aldosterone system inhibitors, other antihypertensive treatment, and discontinuation of medication at baseline examination.
